# Structured Pro-Active Care for Chronic Depression by Practice Nurses in Primary Care: A Qualitative Evaluation

**DOI:** 10.1371/journal.pone.0075810

**Published:** 2013-09-12

**Authors:** Madeleine Bennett, Kate Walters, Vari Drennan, Marta Buszewicz

**Affiliations:** 1 Research Department of Primary Care & Population Health, University College London, Royal Free Hospital, London, United Kingdom; 2 Faculty of Health & Social Care Sciences, St. George’s University of London & Kingston University, London, United Kingdom; The University of New South Wales, Australia

## Abstract

**Purpose:**

This qualitative study explored the impact and appropriateness of structured pro-active care reviews by practice nurses for patients with chronic or recurrent depression and dysthymia within the ProCEED trial.

ProCEED (Pro-active Care and its Evaluation for Enduring Depression) was a United Kingdom wide randomised controlled trial, comparing usual general practitioner care with structured ‘pro-active care’ which involved 3 monthly review appointments with practice nurses over 2 years for patients with chronic or recurrent depression.

**Method:**

In-depth interviews were completed with 41 participants: 26 patients receiving pro-active care and 15 practice nurses providing this care. Interview transcripts were analysed thematically using a ‘framework’ approach.

**Results:**

Patients perceived the practice nurses to be appropriate professionals to engage with regarding their depression and most nurses felt confident in a case management role. The development of a therapeutic alliance between the patient and nurse was central to this model and, where it appeared lacking, dissatisfaction was felt by both patients and nurses with a likely negative impact on outcomes. Patient and nurse factors impacting on the therapeutic alliance were identified and nurse typologies explored.

**Discussion:**

Pro-active care reviews utilising practice nurses as case managers were found acceptable by the majority of patients and practice nurses and may be a suitable way to provide care for patients with long-term depression in primary care. Motivated and interested practice nurses could be an appropriate and valuable resource for this patient group. This has implications for resource decisions by clinicians and commissioners within primary care.

## Introduction

Chronic and recurrent depression are very common [1,2] and are often solely managed in primary care[3]. At least 50 percent of patients who experience major depression are likely to experience further episodes [4,5] but, despite their increased psychological, physical and social morbidity, there is little consistency in the longer term management of these patients[1,6,7].

Structured proactive care is based on key aspects of a collaborative care approach and chronic disease management principles [6]. It aims to provide anticipatory pro-active contact and follow up by a case manager, in this case practice nurses, supported by General Practitioners (GPs, also known as family physicians) within primary care. The collaborative care approach was developed in the United States (US) as a way to improve the management and outcomes of people with acute major depression in primary care[8-10]. Central to this approach is the role of the case manager who proactively follows up patients, assesses adherence to psychological and pharmacological treatments, monitors their progress and takes action when treatment is unsuccessful. This approach has been shown to be effective, with increased remission rates and adherence to treatment, improved depressive symptoms and good economic value[11-14]. In contrast to the US approach in which combinations of three distinct professional groups work collaboratively in a primary care setting (i.e. case manager/practice nurse, primary care practitioner and mental health specialist) [8-10] the ProCEED trial focused on the practice nurse as a case manager within a chronic care model. This is similar to their widespread role within the United Kingdom (UK) in monitoring and reviewing other long term conditions such as diabetes and COPD, and could therefore be more easily implemented in primary care settings using existing staff.

Studies in the US have shown that nurses can be effective case managers for depression[15-17]. Practice nurses in the UK are employed by GPs to work in their practices as part of the primary healthcare team. They are at minimum Registered Nurses (RNs), usually with substantial nursing experience and some may have a specialist qualification in practice nursing [18], although it is not a formal requirement. A minority are also Registered Mental Health Nurses (RMHN), but most will have only received some theoretical background and short clinical placements in mental health settings during their RN course. The role of practice nurses has expanded significantly over the last twenty years as the management of long term conditions has moved away from secondary care. They commonly provide effective, on-going care of patients with long term physical conditions such as asthma, diabetes and hypertension in the primary care setting[19]. In this work, they complement and support the work of the GPs as part of the team, rather than acting as substitutes[20]. Practice nurses in the UK may therefore be well placed to provide a case manager role for chronically depressed patients and may be a valuable resource for the management of this patient group.

Our study was a qualitative exploration, drawing on an interpretative approach [21], of the experiences of both patients and practice nurses within the Pro-active Care and its Evaluation for Enduring Depression trial (ProCEED). This was a national randomised controlled trial comparing usual GP care with anticipatory ‘pro-active care’ and involved 558 participants fulfilling DSM-IV criteria for chronic major depression, recurrent major depression and chronic dysthymia, recruited from 42 General Practices across the UK [6]. The methodology and full trial results are reported elsewhere[6,22]. The aim of this paper is to explore both patients’ and practice nurses’ perspectives on their experience of receiving and providing proactive care. This qualitative data is very important when considering the acceptability and generalizability of such an approach.

### Study Setting – The ProcEED Randomised Controlled Trial (RCT)

The ProCEED pro-active care intervention consisted of an initial consultation with the practice nurse and a further two appointments in the next three months, followed by three monthly reviews for 21 months. Patients had the option to elect for telephone appointments when attending the surgery was difficult, but they were encouraged to attend the surgery for their appointments where possible and most appointments were face to face. If clinically indicated the reviews could be more frequent and if nurses were concerned about a patient, they were asked to discuss them with the relevant GP, who might also see the patient if indicated.

At each review session the practice nurses addressed their patient’s current mood, social circumstances, treatment options and concordance with agreed treatment. After receiving specific training they were encouraged to explore the individual patient’s needs and provide appropriate educational materials and referral to other agencies if required. Whilst doing this they also facilitated participants to identify their own problems, solutions, motivations for change and preferences for care.

The nurses received three full days training, encompassing an overview of pharmacological and psychological therapies for depression and basic training in motivational Interviewing and problem-solving techniques, along-side case-based discussion of their more complex and challenging participants. Each nurse was assigned to one of three clinical supervisors with whom they had regular telephone contact every three to four months, with additional contact if requested.

## Methods

### Design

Qualitative in-depth interviews.

### Study population and sample

All the practice nurses participating in the trial were initially approached to take part in this additional qualitative component. The study was publicised at the ProCEED practice nurse training days and each nurse was sent an invitation letter containing a reply slip and a study information sheet. 40 practice nurses were approached, with 30 agreeing to take part in the interviews; one nurse did not respond.

Research governance approvals were obtained for the Primary Care Trust (PCT) areas where consenting nurses were located. Patients in the trial intervention arm in these geographical areas were then approached to participate, both participants linked to practices where the nurses had agreed to be interviewed and also from other ProCEED practices within these PCTs. This was in order to obtain linked data between nurses and patients, but also to exclude any potential bias if only patients linked to practices where the nurses had agreed to be interviewed were sampled.

Patients were sent an invitation letter containing a reply slip and a study information sheet. Seventy nine patients were contacted, of whom 59 responded with 48 people agreeing to take part. Participating patients were then purposively selected according to their type of depression, i.e. chronic major depression, chronic dysthymia or recurrent major depression, as well as level of attendance at pro-active care review sessions and whether their nurse had agreed to take part in the qualitative interviews. When recruiting patients we also aimed to ensure diversity in demographic characteristics (age, gender, ethnicity).

The practice nurses were purposively selected to represent a spectrum of attitudes using baseline Depression Attitude Questionnaire (DAQ) [23,24] responses, experience and confidence in the management of depression (based on a questionnaire administered at baseline), and ProCEED trial patient dropout rates of participants over the 21 month follow-up. We recruited both nurse and patient participants until saturation on key themes was reached [25]. Saturation was defined as the point in data collection when new data produced little or no change to the thematic framework process and analysis.

### Data Collection & Analysis

All interviews were conducted by the same researcher (MBe), audio-recorded with consent and field notes taken; they lasted on average one hour. Interviews with the practice nurses were carried out at the GP surgery, whilst patients were given a choice of being interviewed either at their home or GP surgery. Interviews were structured around two separate topic guides for patients and practice nurses. These were developed in line with the main aims of the initial research proposal, the background literature and results from the feasibility study for the ProCEED trial, piloted and amended.

The practice nurse topic guide explored in detail how they had conducted the sessions, the usual content, the approach they employed and their experience of providing such mental health review sessions within their clinical practice. We also explored their perceptions of the impact of this approach on patients, as well as the clinical and personal impact on the nurses themselves i.e. confidence, clinical skills, attitude and knowledge. They were asked to give their views about the training and support they had received over the two years, how they thought this approach compared with routine primary care for depression and the practicalities of proactive care for chronically depressed patients in primary care e.g. time allocation, ensuring patient attendance etc.

The topic guide developed for the patients explored their experiences of proactive care, how they felt the case manager approach had impacted on them and if there had been any perceived effect on their depression. We also explored what the patients saw as the nurses’ role within the sessions and how they felt this compared to care provided by other healthcare professionals.

All interviews were transcribed and the data analysed using the framework analytical process [26]. This approach was used as it enabled us to systematically explore the data whilst maintaining an effective and transparent audit trail, in order to enhance the rigour and credibility of our findings. All stages of the framework analysis process were adhered to, with familiarisation and immersion in the data allowing key issues and concepts to be identified. At least two members of the team reviewed each transcript independently and a thematic framework was developed and agreed by consensus. Themes were developed from emerging data rather than the topic guides. The transcripts were then charted using Excel spreadsheets. These charts were used to define concepts, create typologies and provide explanations for the findings. The data was analysed comparatively and disconfirming evidence searched for; the analysis and interpretation remained grounded in the data at all times.

The researcher who conducted the interviews (MBe) was a trainee GP. To limit this potential bias, where possible, this clinical role was not divulged to either the patients or practice nurses who took part in the interviews unless specifically asked; this only happened on one occasion with a practice nurse prior to the interview being completed. To further foster reflexivity in the research process several researchers were involved in the analysis process. The research group consisted of clinical researchers with backgrounds in bothprimary care and nursing, as well as a service user representative. MB and KW were involved in the clinical trial and supervision of nurses but MBe and VD were independent of the ProCEED study. This allowed the development of complementary and divergent understandings and analysis of the data.

### Ethics statement

This study received ethical approval from the Royal Free Hospital & Medical School Research Ethics Committee as a significant amendment to the main trial protocol on 20^th^ May 2009. REC reference number 07/Q0501/15.

A detailed study information leaflet was posted to each participant with confirmation of the interview date at least 24 hours before. At interview the participants had the opportunity to discuss any questions which may have arisen after reading the study information sheet and then to decide whether they agreed to take part. Full informed written consent was obtained by the researcher before proceeding to the qualitative interviews.

## Results

### Sample characteristics

Fifteen practice nurses and twenty six patients were interviewed. The practice nurse characteristics are shown in Table 1. Ten of the nurses were seeing patients regularly in a clinical practice nurse capacity, the other six were full time research nurses. The nurses were drawn from 15 different practices nationally and the patients from 18 practices. The patients’ socio-demographic characteristics are shown in Table 2.

**Table 1:** Practice nurse characteristics.

Practice nurse characteristics	In qualitative study n=15 (%)	In PROCEED n=42 (%)
Full time clinical	7	
Part time clinical and research	3	
Full time research	5	
Years practice nurse experience	3-30yrs	Data not available
Number of intervention participants	5-12	2-12
Drop out rate of participants	0-50%	0-60%
Number of nurses with high participants attendance rates	10/15 (66%)	26/42 (62%)
Number of nurses with low participant attendance rates	5/15 (33%)	16/42 (38%)

**Table 2:** Patient characteristics by diagnosis.

Patient characteristics	Chronic major Depression n=9 (%)	Recurrent Depression n=10 (%)	Chronic dysthymia n=7 (%)	Total n=26 (%)
Female	4 (44.4)	4 (40)	4 (57.1)	14 (53.8)
Male	5 (55.6)	6 (60)	3 (42.9)	12 (46.2)
Mean age (range)	56.3 (39-78)	51.3 (33-71)	61.5 (41-73)	55.8 (33-78)
Ethnicity - white	8 (88.9)	9 (90)	7 (100)	24 (92.3)
Ethnicity - other	1 (11.1)	1 (10)	0 (0)	2/26 (7.7)
Married/cohabiting	5 (55.6)	7 (70)	4 (57.1)	16 (61.5)
Divorced separated/ widow/ single	4 (44.4)	3 (30)	3 (42.9)	10 (38.5)
Attended all sessions	4 (44.4)	3 (30)	3 (42.9)	10 (38.5)
Missed one session	1 (11.1)	1 (10)	0 (0)	2 (7.7)
Missed >2 sessions	2 (22.2)	4 (40)	3 (42.9)	9 (34.6)
Missed more than half Sessions	2 (22.2)	2 (20)	1 (14.3)	5 (19.2)

Three main themes emerged from the thematic analysis: (1) The experience of proactive care reviews, (2) the therapeutic alliance and (3) practice nurses as case managers for depression. These themes enabled us to identify factors which impacted on whether pro-active care was likely to work well, and where it was likely to be more problematic. These themes are discussed below.

### (1): The experience of proactive care reviews

Both nurses and patients identified several key aspects of the experience of the proactive care reviews, including the regular structure and timing of the proactive reviews, the professional impartiality provided by the nurses and improved patient self-awareness of their depression.

#### The structured timing of the proactive reviews

The structured format of the proactive reviews allowed the patients to receive continuity of care with one healthcare professional at their GP practice over a prolonged time period. In addition to this, the patients recognised and appreciated the reviews as a regular designated time for them to discuss only their mental health concerns. Most patients identified this as an important factor in allowing them to talk openly and honestly about their depression with the practice nurses.

I think I was glad in a way because she was specifically for that whereas other things it’s not always specific. And if you go and go to the doctor or anything you talk about umpteen different things but this was specific. I think that was all helpful really (Patient 15).

*It helped seeing them regularly because you got to know what their lives were about. And I think until you’d built up that rapport the first few were a bit staid and I think once they sort of opened up a bit more and talked a bit more about how they were feeling and coping* (Nurse 14).

#### The professional impartiality of the nurse

Most patients reported that the process of talking openly to someone professional and impartial about their depression was helpful, as it allowed them to access objective, constructive feedback. This was mirrored in the practice nurse data, where many nurses reported being able to help patients identify and acknowledge potential triggers for their depression.

*So when you talk to somebody it’s an opportunity to actually discuss some things and see them from a different perspective. Because they have a different slant on it. And sometimes you make out an issue to be quite big, when it’s actually quite minor. You put it into perspective* (Patient 20)

*Once she sort of got more of an understanding of how she was looking at herself and it was more a confidence thing, and that people weren’t criticising her and judging her all the time, she actually could see a way forward. So each session got better with her* (Nurse 14)

#### Improved patient self-awareness

The constructive feedback provided by the nurses during the review sessions enabled some of the patients to develop a greater understanding and self-awareness of their depression. When this occurred it was reported by both patients and nurses as providing a base from which the patients were able to recognise and address the impact of their mental health on their wider physical and social situation.

*The talking about my depression openly, the problems that I’ve had, what we can do to solve those problems, any goals that we can do, any tasks that we can do to actually solve those. So I think it’s just been a big learning curve for me to pick up tips, as I say to treat something which is going to be a chronic condition and I’ve just got to learn to manage it the best I can (Patient 17*)

*It’s confidence for the patients. I think it’s confidence, always that they understand more their condition and how to manage it and I think that must benefit the patient and reduce GP time* (Nurse 11)

### (2): The therapeutic alliance

The role of the case manager was not intended to include that of therapist, but in the interviews a therapeutic relationship which could be termed a form of ‘therapeutic alliance’ was described by both patients and nurses, and this appeared to have an important impact on their experiences of proactive care. The development, or conversely lack of development, of such an alliance was affected by a number of factors, such as the clinical approach used by the nurses within the review sessions and the ability or willingness of both the patients and nurses to engage in the process. We have described these further under nurse and patient factors below.

#### Nurse factors

Interviews with both nurses and patients demonstrated that, despite all attending the same training sessions, the nurses varied in the way in which they delivered proactive care to their patients. We identified four different approaches the nurses used with their patients, some nurses using more than one approach, as below:

I. Non-directive counselling: this would incorporate ‘active listening’ and appeared to be used by the nurses especially at times of crisis for the patient
II. Problem solving: this might be led by the patients, facilitated by nurses or more directly led by the nurses within the review sessions
III. Facilitation: this involved actively helping the patient to navigate access to other support structures e.g. seeing their GP, community mental health team (CMHT) or other services such as voluntary work, debt relief agencies, enrolling on courses of interest.
IV. Protocol driven approach: this approach was concerned with completing the study documentation, going through study formalities and questionnaires and was often perceived by the patients to be more disengaged and potentially alienating.

The approaches used by the nurses seemed, at least in part, to be associated with their previous clinical experience and interest in mental health, as well as their level of comfort with mental health issues. Nurses who seemed more confident and comfortable with mental health issues at the start of the trial appeared to include a stronger emphasis on a more therapeutic, counseling approach, whereas others who were not so confident at baseline favoured a more directive, protocol-led approach similar to the style used in their regular clinics which could be difficult for the patient to engage with. Examples are given below.

Non directive counseling: *‘She doesn’t talk about it but I might and she’ll reflect it back at me, a bit like a psycho-analyst might but without the depth. It doesn’t need to be deep. I don’t need to go into all the horrors of the past. So it sort of feels – it feels as those she’s walking beside me rather than – like the behaviourists always seem to be walking in front trying to show you the way’.* (Patient 10)

Problem Solving: *‘I think it was the fact I was asked what the problems were and I, you know, I was at that moment in time able to say them. And she worked through them very good, very progressively, it was quite, it had order to it. I thought that went very well’* (Patient 8)

Facilitation : *‘it was nice just being, almost to sit here as a signpost so people could come in with their problems and you could point them sort of in which way to go, sort of who could help them if* *I* *couldn* ’*t, and refer them on either for counselling, or point them to Mind’* (Nurse 2)

Protocol driven: *‘Sit down. Right, I’ve got to read this bit out for you and then we’ll answer the questions.’ Which makes you feel that you don’t want to chat to them anyway. Because I felt that I was taking up their time. It was the way it came across’* (Patient 16)

Most patients reported a positive experience with the first three approaches, although the more focused nurse led problem solving approach could lead to conflict between patient and nurse when suggestions and goals weren’t attempted or achieved by the patients, leading to frustration on the part of the nurse. The protocol driven approach was more often perceived by patients to be less personal, more detached and more likely to hamper the development of a therapeutic alliance.

#### Nurse motivation and interest

A few of the practice nurses were perceived by patients interviewed for the qualitative study as reluctant and lacking motivation. When this occurred the patients reported feeling that the nurse didn’t appear at ease with mental health issues and gave an impression of being coerced to provide the intervention, leading to them feeling uncomfortable during the review appointments. Mirroring this sentiment, the three nurses who seemed to gain least from taking part in the ProCEED trial described themselves as not particularly interested in mental health and, rather than choosing the project themselves, as having been encouraged to take part in the trial by an interested GP within the practice. These nurses appeared more reticent about taking part and felt less positive about their experience and what they had gained from being involved the trial over the two year time period.

*I was quite reticent about doing it, because I haven’t really had a huge interest in it* (Nurse 3)

#### Nurse typologies

By recognizing the above factors we have developed typologies to describe the practice nurse approaches, as detailed below.

1 The Counselor: These nurses were more comfortable with mental health issues and had often had more positive experiences of working with patients with mental health problems through their previous training and clinical experience; they would use a mainly non-directive counseling style approach and facilitated the sessions according to the patients self-recognized needs. A strong therapeutic alliance was often developed with these nurses and patient attendance rates were higher.
2 The Facilitative Problem Solver: These nurses were also generally more comfortable with mental health issues. They would adopt a problem solving approach and actively facilitate the participant to find solutions and make changes in their lives. Some nurses would blend this with a non-directive counseling approach at times of crisis for the participant.
3 The Directive Problem Solver: These nurses were less comfortable with mental health issues and would incorporate a more protocol driven consulting style in to the reviews. They would use a combination of problem solving and facilitation approaches to identify areas which needed addressing and tended to present the patient with the solution to their problems. Some patients responded well to these nurses, others found the approach too directive. These nurses voiced the most frustration when the patients did not respond or act on their suggestions.
4 The Reluctant Nurse: These nurses were reluctant from the start, were less keen to take on the project and less comfortable around patients with mental health problems. They were the least likely to use the non-directive counseling approach and would be more likely to be more protocol driven. Patients found it harder to develop a therapeutic alliance with these nurses and patient attendance levels were lower.

#### Patient factors

The motivation and engagement of the patients also emerged as key in the development of both a therapeutic alliance and perceived positive outcomes from the proactive care intervention.

#### The engaged patient

Whether a listening or more directive approach was used by the nurse they saw, the ability of the patient to engage with the process appeared to also impact both on their experience and that of the nurse over the two year period. Patients who seemed to make the most progress over this time and to report a positive experience were those who attended the majority, if not all the sessions, appeared to carefully consider the suggestions and signposting provided by the nurses and made lifestyle changes as a result. If the patients were perceived to be motivated, open and honest the nurses felt that they ‘wanted to get better’ and this appeared to contribute to a positive therapeutic alliance.

*I kept thinking, I’m gonna beat this, I’m gonna beat this, and I was* (Patient 11).

*She came back and said to me, ‘Right I’ve got help coming in. I’ve done this. I’ve done that. I feel so much better. I’m going to do this and that.’ And I sort of thought gosh you know, and I just felt – I hope together, you know both working together this woman has changed, this patient has changed so much in sort of gaining some confidence and being able to think about herself and manage the situation* (Nurse 11)

#### The reluctant patient

The relationship between the patient and nurse was hampered if one party was perceived to lack motivation or be reluctant to take part. Patients who seemed to get less from the intervention had lower attendance rates, attending as little as two sessions in the 2 year period, or had multiple telephone rather than face to face sessions. Initial poor patient-nurse interaction, lack of motivation and time, a reluctance to talk to the nurse about their difficulties, or a lack of belief that their situation would change were cited as factors in poor attendance.

Y*ou get out of it what you put into it, and the way I am I probably don’t put much into it. It’s hard to tell people how you feel or why you feel, or why you do this and why you do that. And I probably don’t* (Patient 25)

*He is someone that whatever you suggest it makes no difference at all. And in fact* *Ilearnt* *very early on that it wouldn’t matter if we gave him the world actually. He says no to everything. He’s not prepared to do anything. He’s not pro-active. And actually I think he likes living the way he does* (Nurse 5)

### (3): Practice nurses as case managers for depression

This theme describes the particular skills which the patients attributed to the practice nurses during their review sessions and considers how these developed over their two years as case managers.

#### Nurse led care

Most patients reported having strong preconceptions about the particular qualities of practice nurses including how this contrasted with their GPs in providing care for people with depression. The majority of those interviewed felt that discussing their depression with their GP wasn’t appropriate, unless it was for a distinct reason such as medication or concurrent physical symptoms. By comparison most felt more prepared to discuss their mood and wider social concerns with the practice nurses.

I couldn’t sit and talk to Doctor H*****, I mean not that he would ever, ever give me the impression that he wanted me out and moving on. He’s wonderful. But I didn’t want to be wasting his time by rabbiting on about things (Patient 9)

*You’re a nurse, you’ll understand me. I don’t want to bother the doctor with this.’ I mean that’s their opening line* (Nurse 1)

#### Active facilitation

Many patients felt that the nurses’ role within the practice was important to their clinical care, including their ability to liaise with the GPs, arrange appointments and ensure quick access to a GP if the nurse felt this was needed was important to their clinical care. In some cases the nurses were able to discuss patients with counsellors in the practice, and some nurses developed links with the local CMHT, aiding referrals and communication with secondary care services.

She’s been worried enough about me to say, ‘Before you leave here I want you to have made an appointment to see Dr H*****, and has checked that I have done so, which is a safety net (Patient 10).

#### Information giving

The practice nurse was also able to provide information regarding medication and health education, improving patients’ understanding of both their physical and mental wellbeing and how the two might interact.

*I mean that’s where I’m grounded basically do you know what I mean, doing the healthy lifestyle bit. So yes it was very much part of the interviews which was something they all wanted to discuss anyway because weight and appearance and all the rest of it goes very much with the general self esteem picture* (Nurse 12).

#### Sign-posting

Some nurses actively sign-posted patients to services both within the GP practice and also in the wider community. They would guide patients to services which could cater to their individual needs; these ranged from Citizen Advice Bureau and debt relief agencies to local community groups and leisure centre programmes. Where appropriate sign posting had occurred and patients had acted on the suggestions, both they and the nurses reported a positive outcome. Sometimes if patients had not followed up the suggestions made, this could lead to frustration for the nurse and resultant disharmony in the consultation.

## Discussion

This qualitative exploration of both patient and nurse experiences highlights the potential role that practice nurses may be able to play in providing on-going pro-active care for patients with recurrent and chronic depression. Central to this model of care was the ability of the patients and nurses to develop a ‘therapeutic alliance’ within the review sessions. Where this appeared lacking dissatisfaction was felt by both patients and nurses, with a potentially negative impact on outcomes.

The importance of the therapeutic alliance, the collaborative and affective bond between therapist and patient [26], has previously been recognised as an essential element of the therapeutic process in working with patients with psychological problems, with reviews consistently finding that the quality of the alliance between practitioner and patient is related to subsequent therapeutic outcome[27]. Our study adds to this evidence by triangulating patient and practice nurse data to develop a more coherent understanding of the factors which may help or hinder the development of such an alliance.

Motivation and reluctance on the part of both nurses and patients were important themes in the development of a therapeutic alliance. By assessing the typologies of the nurses involved we found that nurses who were less confident with mental health issues generally, or who reported a previous poor experience with people with mental health problems, tended to follow a more protocol driven, detached approach to the review sessions and found it difficult to develop a therapeutic alliance with the patients. This emphasises the importance of recruiting a skilled and empathetic case manager when working with people with mental health problems [27] and complements other studies which indicate that if a mechanical ‘tick box’ approach appears to be being used then case management for mental health problems can be experienced by patients as less beneficial[28,29]. A previous qualitative assessment of case managers within mental health based collaborative care studies has also highlighted the importance of interpersonal skills[30]. Practice nurses can also find it difficult to provide lifestyle counselling if they consider themselves deficient in the appropriate skills or lack motivation themselves[31]. It is therefore critical that if pro-active care is to be provided by practice nurses in primary care settings that feel confident in their ability to address patients’ individual needs and that they receive adequate training to ensure an empathetic and appropriate response[29,32].

Patients perceived practice nurses to be suitable case managers for the management of their mental health problems if the nurse was experienced as understanding, caring and approachable. In addition, the interview data provides evidence of patients’ preconceptions about the roles of both the GPs and practice nurses within primary care. Most patients reported uneasiness in reviewing their mental health problems with their GP and a willingness to be more open with the practice nurse by comparison. Existing literature supports these preconceptions and has shown that patients are uncertain whether mental health reviews in primary care are a legitimate use of the GP’s time, and whether they will be listened to and understood by them[33-35]. Within our study it appeared that the patients perceived the practice nurses as effectively and appropriately complementing the work of the GPs in this area[20].

The acceptability of practice nurses as case managers provides further scope for an on-going discussion about the role of practice nurses in complementing the work of GPs [20] in primary care. Practice nurses commonly deliver healthcare management to people with long term conditions and a recent meta-analysis of nurses within a case manager role for both mental and physical conditions has shown clinical effectiveness with good long term follow up outcomes[15]. In addition, the recent expansion of the case manager role to provide care for depression in conjunction with other common comorbid conditions such as diabetes and hypertension in recent trials would appear to provide support for practice nurses to provide this role[15,17,36,37].

The strength of this research is that it allows us to compare both patient and nurse experiences and, by triangulating the data, we are able to provide a comprehensive analysis of aspects of the intervention which were both beneficial and unacceptable to the patient and the care giver. This is reinforced by the strong correlation between the patient and practice nurse data, with no conflicting data in the in-depth interviews. The main limitation is a potential sampling bias, since more motivated patients and nurses who participated in ProCEED may have agreed to take part. We however purposively sampled to ensure a broad range of views, attendance rates and experiences, and included patients with low attendance rates and nurses who reported low baseline confidence levels, more negative attitudes towards depression or who had higher drop-out rates of intervention patients. The researcher included probes in all the interviews for negative experiences and perceptions.

Our study has identified that motivated and interested practice nurses can be an appropriate and valuable resource in the management of patients with chronic and recurrent depression in the primary care setting. With the current emphasis on moving the management of chronic and long-term conditions into primary care, and the expansion of the case manager role to include the management of comorbid conditions such as diabetes [15,36,37], this study has important implications for the reorganisation of care and resource decisions by both clinicians in general practice and commissioners within primary care. A longer term implication of this research relates to its capacity to inform and advocate for the further development of the role of practice nurses within primary care.
